# Radiological Features of Giant Cell Tumours of Bone

**DOI:** 10.7759/cureus.8793

**Published:** 2020-06-24

**Authors:** Emma L Howard, Jonathan Gregory, Naomi Winn, Adrienne Flanagan, Paul Cool

**Affiliations:** 1 Orthopaedic Oncology, Robert Jones and Agnes Hunt Orthopaedic Hospital NHS Foundation Trust, Oswestry, GBR; 2 Orthopaedic Oncology, The Royal Orthopaedic Hospital NHS Foundation Trust, Birmingham, GBR; 3 Radiology, Robert Jones and Agnes Hunt Orthopaedic Hospital NHS Foundation Trust, Oswestry, GBR; 4 Pathology, Royal National Orthopaedic Hospital, London, GBR; 5 Medical Sciences, Keele University, Keele, GBR

**Keywords:** origin, giant cell tumour of bone

## Abstract

Introduction

The aim of this study was to evaluate radiological measurements to establish the origin of giant cell tumours of bone.

Methods

A multi-centre retrospective review was conducted of patients with histologically confirmed giant cell tumours of bone. Images were analysed to estimate the centre of the tumour. Measured from the joint line, the ratio between the distance of the centre of the tumour and the physeal scar was calculated.

Results

Ninety-five patients were included in the study. Two observers found the tumour to be arising from the metaphyseal area in 94% - 97% of the cases. There was good agreement between the measurements of observers (interclass correlation coefficient 0.71).

Conclusion

Giant cell tumours of bone appear to be arising from the metaphyseal region.

## Introduction

Giant cell tumours of bone (GCTBs) are intermediate, locally destructive tumours, accounting for approximately 5% of all primary bone tumours. Typically, young adults between the ages of 20 and 40 years are affected, with a predilection for females [1]. Malignant giant cell tumours of bone, albeit rare, have been described, either as primary, where sarcomatous changes are present within otherwise conventional GCTBs, or as secondary where a high-grade sarcoma occurs at a previously treated GCTB site [2]. In approximately 1%-4% of all cases, the development of pulmonary metastases occurs [3]. There is no consensus on factors that increase the likelihood of pulmonary metastases occurring. However, some authors propose that local recurrence is a risk factor for lung metastasis [4-9]. The literature generally reports giant cell tumours of bone to be epiphyseal originating tumours [1]. However, some authors suggest that GCTB may, in fact, originate in the metaphysis [10-13]. Our clinical impression also favours a metaphyseal origin of GCTB.

Giant cell tumours of bone are primarily treated with surgical resection, either by intralesional curettage or wide resection. Intralesional curettage is often favoured, as most giant cell tumours of bone occur peri-articularly and curettage preserves limb function. Some authors favour wide resection to minimise local recurrence. However, these procedures have a higher incidence of surgical complications, including infection, and limited joint function [14]. The management of giant cell tumours of bone is challenged by high rates of local recurrence, which, in part, is a consequence of an intraoperative residual tumour [15]. Denosumab, a monoclonal antibody that binds to receptor activator of nuclear factor-kappa Β ligand (RANKL) and inhibits osteoclastogenesis, can also be used in the management of GCTB. The use of denosumab may be beneficial by reducing the size of the tumour, thereby making surgery technically easier, and may reduce the size of any residual tumour left after surgery [16].

The aim of this study was to evaluate the origin of giant cell tumours of bone on imaging investigations.

## Materials and methods

Patients

A multi-centre retrospective review was conducted of consecutive adult patients with a confirmed histological diagnosis of a giant cell tumour of bone between June 2012 and May 2017 in two primary bone tumour centres. Inclusion criteria included a confirmed histological diagnosis of a non-malignant giant cell tumour of bone and age over 18 years. Patients without appropriate imaging (i.e., severe joint destruction or physeal scar not visible) were excluded.

Magnetic resonance imaging (MRI) images

MRI images were often obtained from outside institutions and the sequences obtained were not standardised. However, as a minimum, a T1 sequence was obtained in all patients. Measurements were taken from the T1 sequence that had a minimum slice thickness of 3 mm.

**Figure 1:**
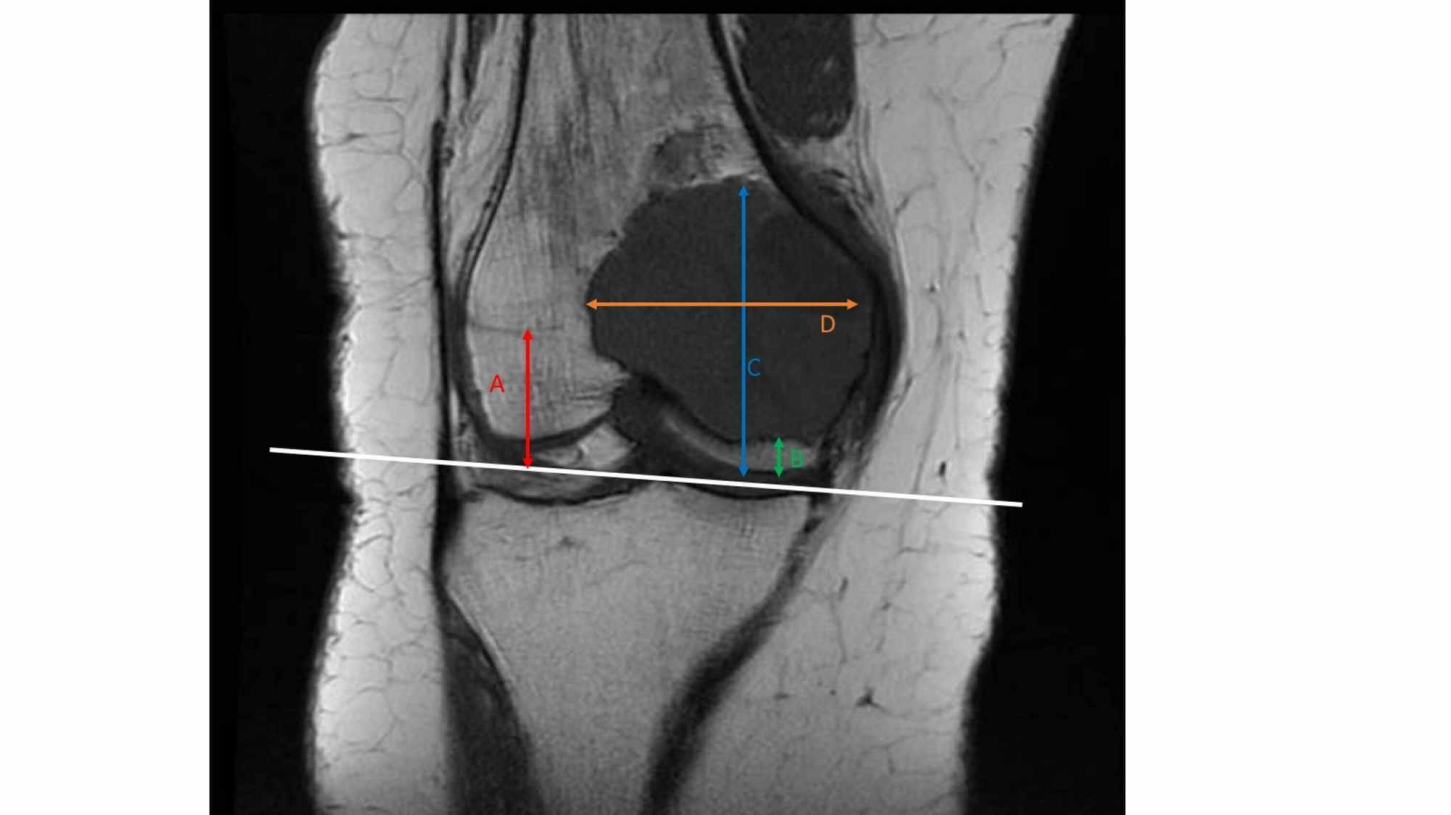
Radiological measurements Coronal T1 MRI scan showing the measurements taken. From the joint line of the affected part of the bone, the distance to the physeal scar (A), inferior margin of the tumour (B) and superior margin (C) of the tumour were measured. The width of the tumour (D) was also measured. All measurements were in pixels. MRI: magnetic resonance imaging

Images were downloaded from the picture archive and communications system (PACS), duplicated into two separate folders, and measured independently by two observers (A and B). Observer A was a consultant orthopaedic oncologist, and observer B was a medical student. The method of taking measurements (Figure 1) was pre-agreed. A medical student was used as an observer to minimise cognitive bias.

Measurements

From the MRI images, the distance from the joint line to the physeal scar was measured (Figure 1A). The distance from the joint line to the inferior aspect of the tumour (Figure 1B) and the distance from the joint line to the superior aspect of the tumour (Figure 1C) were measured. The width of the tumour was also measured (Figure 1D).

**Figure 2:**
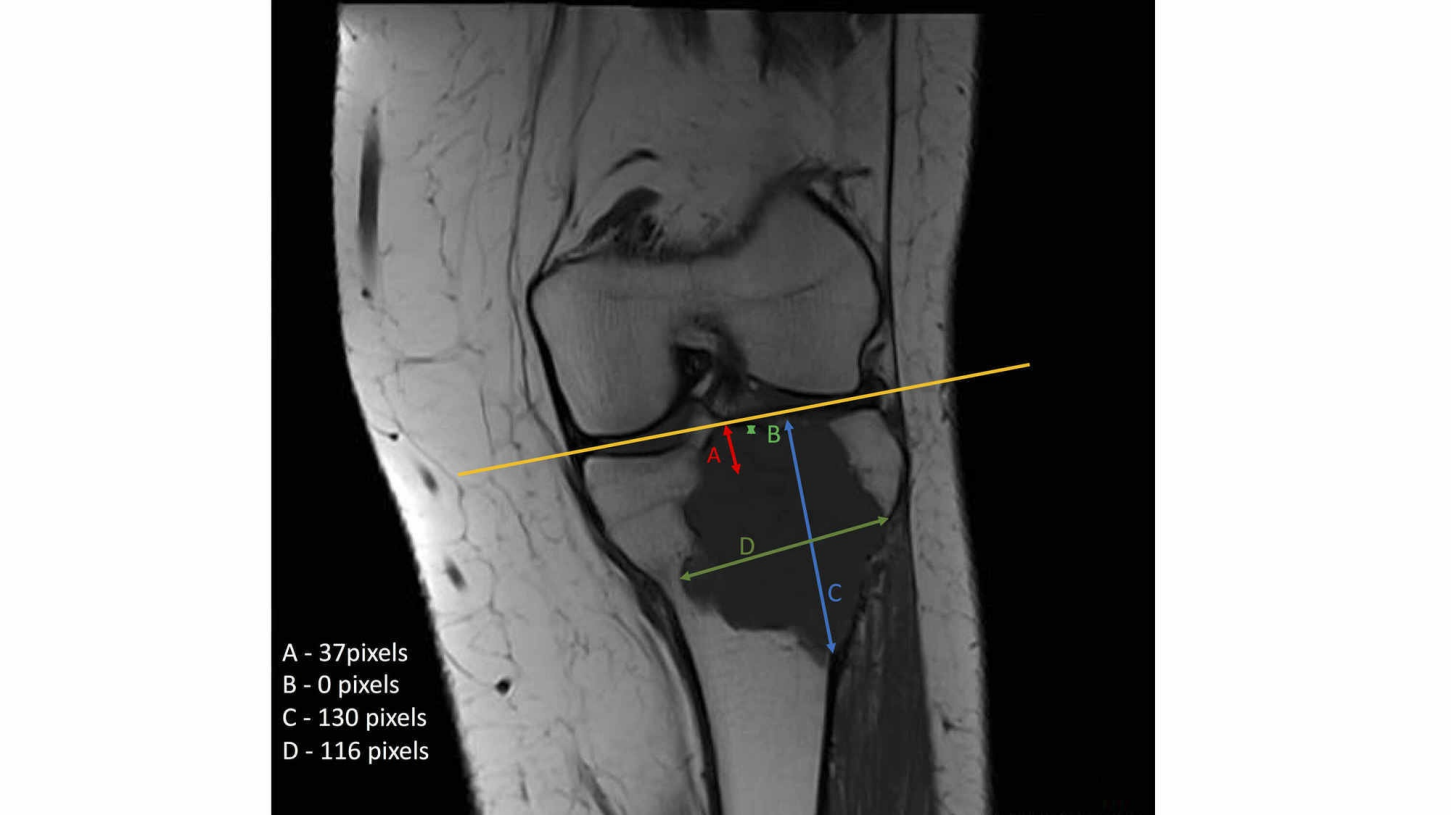
Example calculation of the centre of the tumour and the ratio between the physeal scar and the centre of the tumour The figure depicts the following measurements, which are all taken perpendicular to the joint line (orange line). 2A: the distance from the joint line to the physeal scar, 2B: the distance from the joint line to the inferior aspect of the tumour (from the joint line), 2C: the distance from the line to the superior aspect of the joint line, 2D: the width of the tumour. The length of the tumour was first calculated: (Fig 2C - Fig 2B) 130 - 0. Length = 130 pixels. The centre of the tumour was calculated by dividing the length of the tumour by 2 (65 pixels) and adding it to the distance from the joint line to the inferior margin (2B). So 65 + 0 = 65 pixels. To calculate the ratio between the centre of the tumour and the physeal scar, the centre of the tumour is divided by the distance from the joint line to the physeal scar (2A). Therefore, 65 / 37 = 1.75 and, consequently, this tumour is metaphyseal in origin.

The length of the tumour was calculated by subtracting the distance from the joint line to the inferior margin of the tumour (Figure 1B) from the distance from the joint line to the superior margin of the tumour (Figure 1A). The centre of the tumour, measured from the joint line, was found by dividing the length of the tumour by two and adding it to the distance from the joint line to the tumours inferior margin (Figure 1B). Subsequently, the ratio between the centre of the tumour and the distance to the physeal scar was calculated (Figure 2).

**Figure 3:**
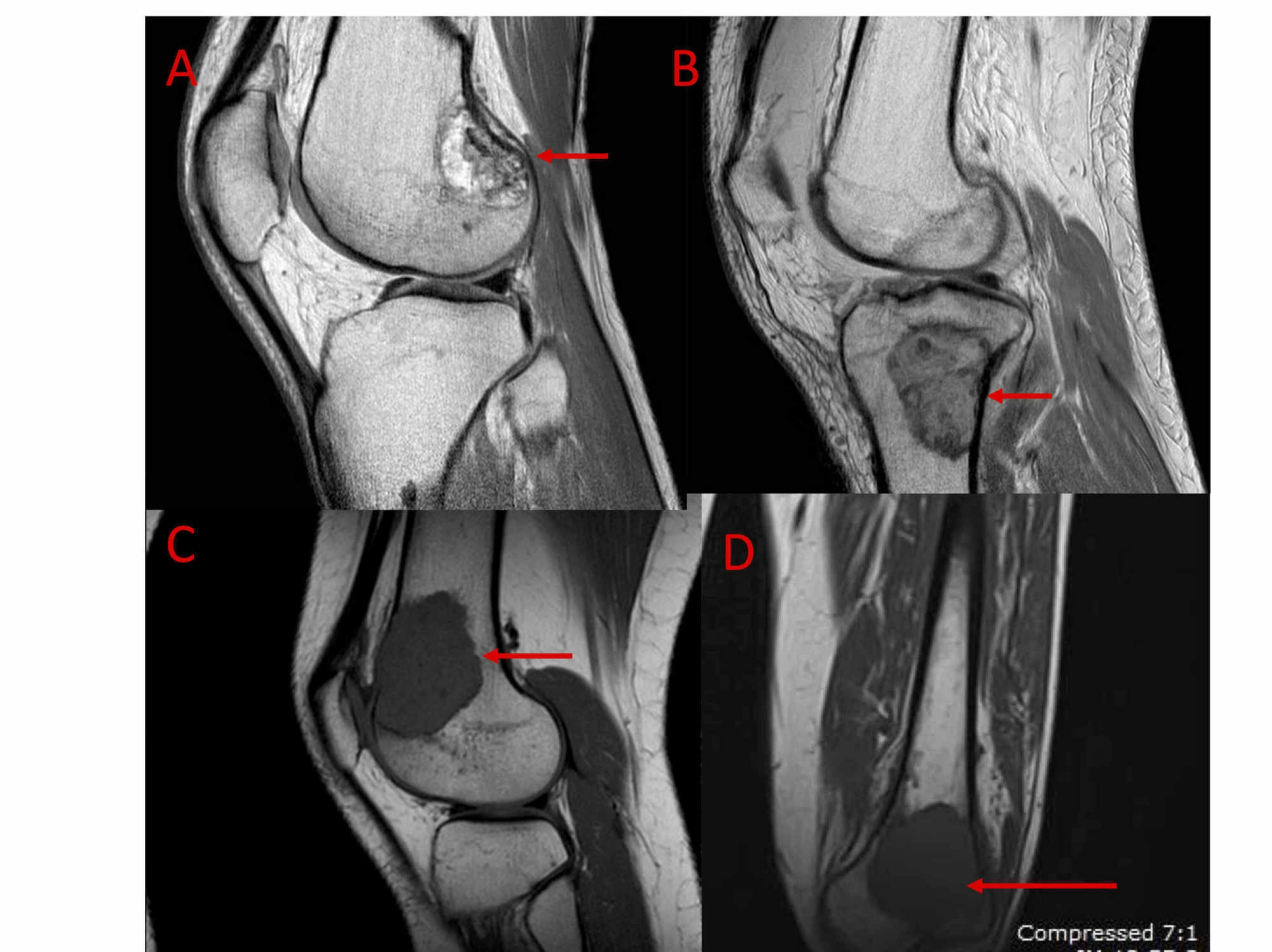
Metaphyseal originating giant cell tumours of bone MRI images showing examples of histologically proven giant cell tumours of bone, (2A), (2B), (2C), (2D), which appear to be predominantly metaphyseal in origin. Red arrows highlight the location of the lesion.

A ratio of less than one indicates the tumour originates in the epiphysis and a ratio of greater than one suggests a metaphyseal origin (Figure 3).

Statistical analysis

Data were analysed with R statistical software (R Foundation, Vienna, Austria). Boxplots of ratios were created and agreement between observers was assessed with the interclass correlation coefficient [17]. Bland-Altman plots with 95% confidence intervals were used to assess agreement and measurement bias.

Interclass correlation coefficient values between 0.40 and 0.59 were regarded as showing fair agreement, between 0.60 and 0.74 as good agreement and greater than 0.75 as excellent agreement.

## Results

Ninety-five patients were identified from a consecutive cohort of 195 patients that had all required imaging available for review. One hundred patients were excluded, as the physeal scar was not visible on images and, therefore, measurements could not be completed. Fifty-six patients were male (59%) and 39 patients were female (41%). The median age was 35 years (interquartile range (IQR): 27 - 47 years). Forty-four (46%) tumours were on the left side and 51 (54%) on the right side. The most common site of tumour was the distal femur in 43 (45%) cases, followed by the proximal tibia in 36 (38%), proximal femur 4 (4%), proximal humerus 4 (4%), metacarpal 3 (3%), distal humerus 1 (1%), distal tibia 1 (1%), proximal fibula 1 (1%), metatarsal 1 (1%) and distal radius 1 (1%).

**Figure 4:**
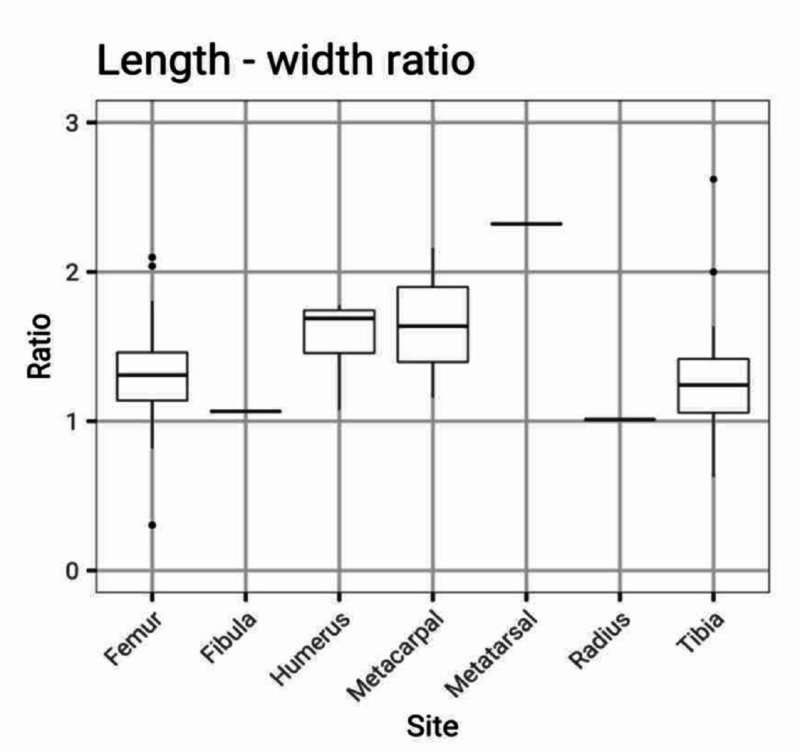
Length to width ratio of giant cell tumour of bone Boxplots to show the ratio between the maximum tumour length and tumour width of giant cell tumours of bone. Horizontal lines represent the median and the vertical lines from the boxes represent the quartiles of data. Dots represent extreme values of data. The tumours were roughly spherical but were slightly longer along the long axis.

Although roughly spherical, most tumours were slightly longer along the long axis of the bone (Figure 4).

**Figure 5:**
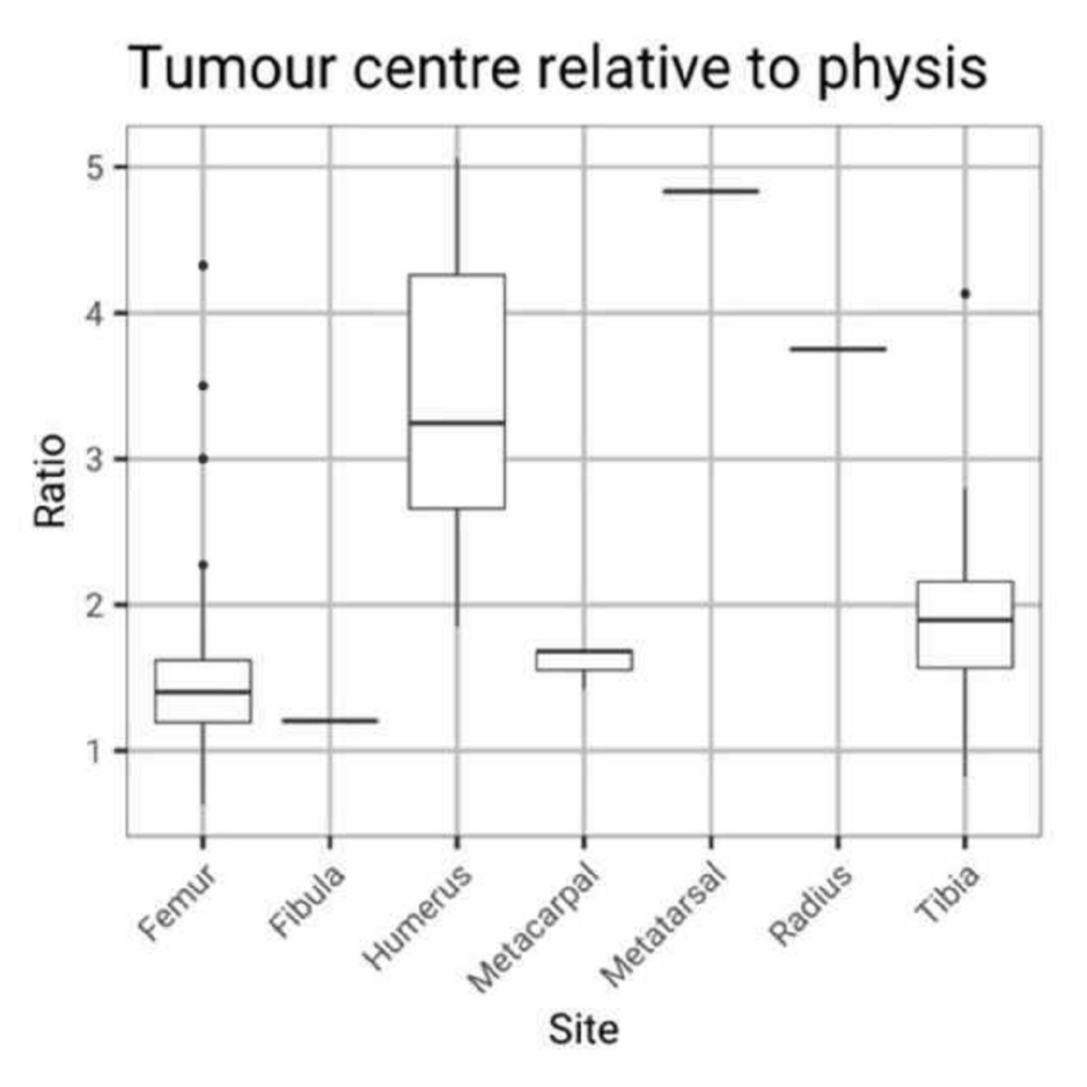
Tumour centre relative to physis Boxplots to illustrate the ratio between the distance of the centre of the tumour to the physeal scar as measured from the joint line. Horizontal lines represent the median of data. Vertical lines represent the quartiles and dots represent extreme values of data. The ratios of the majority of the tumours were greater than 1, indicating the origin of the tumour to be metaphyseal.

The origin of the tumour was found to be predominantly in the metaphyseal area (Figure 5).

**Figure 6:**
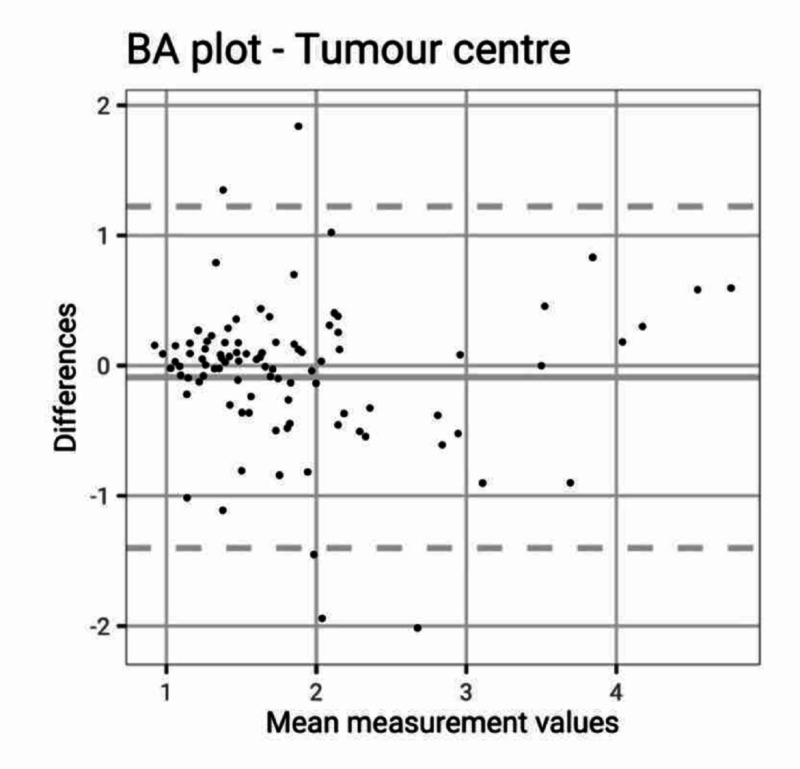
Bland-Altman plot for tumour centre The Bland-Altman plot for the estimation of the ratio between the distance of the centre of the tumour to the physeal scar as measured from the joint line. There was no bias between observers and little variance between observers.

The interclass correlation coefficient for tumour length over width was 0.56 (95% CI: 0.40 - 0.68) showing only fair agreement between observers. However, the interclass correlation coefficient for the ratio of the tumour centre to the physeal scar was 0.71 (95% CI: 0.60 - 0.80) showing good agreement between observers. The Bland-Altman plot also shows good agreement without bias (Figure 6).

Using the ratio as a measure of tumour origin, one observer estimated 97% of the tumours to be metaphyseal in origin whilst the other estimated this to be 94%.

## Discussion

Our study suggests that giant cell tumours of bone are tumours originating from the metaphyseal area. It is generally reported in the literature that giant cell tumours of bone are epiphyseal in origin [1]. Conversely, Murphey et al. suggested that in skeletally immature patients, GCTBs originate in the metaphysis and the open physeal plate acts as a barrier to tumour growth [18]. Consequently, it has been suggested that GCTB may originate in the metaphysis and spread to the epiphysis after physeal plate closure [10]. Although in our study, all patients under the age of 18 were excluded, there was one image measured that had an open physeal plate, which potentially alters the results from that image and is a limitation of our study.

In a recent study by Futamura et al., the investigators calculated the vertical centre of tumours on anteroposterior X-rays of tumours [10]. In their study cohort, 80% of tumours originated in the metaphysis. Our study also supports the metaphyseal origin of giant cell tumours of bone. This opinion is strengthened by the good agreement between observers, irrespective of the level of experience and, therefore, results are also reproducible.

However, measurements are not always easy to obtain. Not all patients had appropriate imaging, and of the 195 consecutive patients, only 95 had suitable imaging available. Most patients with a distal radial tumour had extensive involvement, making measurements impossible, as the physeal scar was no longer visible. Our study only includes one patient with a distal radial giant cell tumour of bone and this may have introduced bias.

Measurements in the distal femur and proximal tibia were usually straightforward to obtain. However, the physis has a more complex arrangement in the proximal humerus, perhaps explaining the larger variance in measurements in that location.

The limited spatial resolution of MRI may have caused further variance in measurements. However, the use of ratios, rather than absolute measurements, helps to reduce this effect.

Our results show that the ratio between tumour length and tumour width is roughly equal, indicating a spherical tumour shape. This seems to indicate equal tumour growth in all directions. Consequently, it appears reasonable to assume a metaphyseal origin as indicated by the measurements.

Tumour growth is supported by osteoclastic resorption of bone in all directions. However, cartilage cannot be resorbed by the osteoclast and tumour growth stops at the subchondral bone. From then onwards, growth can only occur in the width or proximally. Initially, pain may not be a predominant feature. However, once the tumour has reached the subchondral bone, a subchondral fracture is likely to occur, and the patient presents with pain.

## Conclusions

Previously, giant cell tumours of bone were considered to be epiphyseal originating tumours. From our study, measurements from MRI images found giant cell tumours of bone to be roughly spherical, but slightly longer in the long axis, and to be originating from the metaphysis in 94%-97% of cases.
